# Modulation of Intestinal TLR4-Inflammatory Signaling Pathways by Probiotic Microorganisms: Lessons Learned from *Lactobacillus jensenii* TL2937

**DOI:** 10.3389/fimmu.2013.00512

**Published:** 2014-01-14

**Authors:** Julio Villena, Haruki Kitazawa

**Affiliations:** ^1^Immunobiotics Research Group, Tucuman, Argentina; ^2^Laboratory of Immunobiotechnology, Reference Centre for Lactobacilli (CERELA-CONICET), Tucuman, Argentina; ^3^Food and Feed Immunology Group, Laboratory of Animal Products Chemistry, Department of Science of Food Function and Health, Graduate School of Agricultural Science, Tohoku University, Sendai, Japan

**Keywords:** *Lactobacillus jensenii* TL2937, TLR4, intestinal immunity, inflammation, immunobiotics

## Abstract

The intestinal mucosa plays a critical role in the host’s interactions with innocuous commensal microbiota and invading pathogenic microorganisms. Intestinal epithelial cells (IECs) and gut associated immune cells recognize the bacterial components via pattern-recognition receptors (PRRs) and are responsible for maintaining tolerance to the large communities of resident luminal bacteria while being also able to mount inflammatory responses against pathogens. Toll-like receptors (TLRs) are a major class of PRRs that are present on IECs and immune cells which are involved in the induction of both tolerance and inflammation. A growing body of experimental and clinical evidence supports the therapeutic and preventive application of probiotics for several gastrointestinal inflammatory disorders in which TLRs exert a significant role. This review aims to summarize the current knowledge of the beneficial effects of probiotic microorganisms with the capacity to modulate the immune system (immunobiotics) in the regulation of intestinal inflammation in pigs, which are very important as both livestock and human model. Especially we discuss the role of TLRs, their signaling pathways, and their negative regulators in both the inflammatory intestinal injury and the beneficial effects of immunobiotics in general, and *Lactobacillus jensenii* TL2937 in particular. This review article emphasizes the cellular and molecular interactions of immunobiotics with IECs and immune cells through TLRs and their application for improving animal and human health.

## Introduction

The mammalian gastrointestinal tract harbors trillions of beneficial commensal bacteria, a population composed of at least 1,000–5,000 species (1). Studies probing the composition and function of the endogenous microbiota in the normal gastrointestinal tract have greatly expanded our appreciation for an understanding of how the microbiota shape mucosal immune responses, as well as how commensal bacteria in the gastrointestinal tract regulate the production of immunoregulatory, diet-dependent nutrients and metabolites (2). In fact, recent studies have highlighted that alterations in the composition of commensal bacterial populations are linked to multiple metabolic and inflammatory diseases in humans including but not limited to inflammatory bowel disease (IBD), obesity, type 2 diabetes, atherosclerosis, allergy, and colon cancer.

Mammals have an evolutionary partnership with the microbiota that is critical for host defense. In the gastrointestinal tract, part of the local immune response is aimed at maintaining a peaceful coexistence with the resident microbiota. Abundant experimental and clinical data support the idea that commensals residing in the gastrointestinal tract can calibrate both innate and adaptive responses (3, 4). Unique groups of commensals as well as defined metabolites of commensals also can have key roles in the control of mucosal responses (4). Additionally, despite being contained by the intestinal mucosa, the gut microbiota can also modulate immune responses at distal sites in the steady-state and during inflammation (5).

In recent years, the study of microbe-intestinal cell interactions has unraveled several molecular mechanisms and cellular pathways, showing that these interactions play a crucial role in the regulation of several immunological functions in the gut. Moreover, better understanding of the host-microbe interactions in the gut has provided new opportunities for preventing and treating a number of inflammatory disorders such as the use of specific probiotic strains to beneficially modulate the intestinal immune system. Probiotic bacteria that are able to modulate the immune system (immunobiotics) are demonstrably beneficial for treating a variety of mucosal disorders, including inflammatory diseases (6).

Weaning-associated intestinal inflammation occurs in various animal species including the pig. Intensification of the pig industry has brought increased risks of both clinical and sub-clinical enteric disease. Piglets are vulnerable to potentially harmful microorganisms such as *Escherichia coli, Salmonella* spp., and *Clostridium perfringens* (7). Antibiotics have been applied widely in animal husbandry to prevent and treat the gastrointestinal infection caused by pathogens (8). However, the promiscuous use of antibiotics has resulted not only in the emergence and spread of resistant bacteria in humans but also in animals (9). Early weaning of piglets is often accompanied by a high susceptibility to diarrhea. It has been established that this process is multi-factorial and that post-weaning inflammation and malnutrition are major etiological factors. Pigs coexist with a dense and diverse microbiota in their gut. As observed in humans, the microbial colonization of the porcine intestine begins at birth and follows a rapid succession during the neonatal and weaning period (10, 11). Following the withdrawal of sow’s milk the young piglets are highly susceptible to enteric diseases partly as a result of the altered balance between developing beneficial microbiota and the establishment of intestinal bacterial pathogens. In addition to the changes in microbiota composition, the intestinal immune system of the newborn piglet undergoes a rapid period of maturation, expansion, and specialization that is not achieved before commercial weaning (10, 11).

Various nutritional approaches for optimizing the weaning transition and minimizing gut inflammation and enteric diseases have been tested in the past decade. Among the novel dietary strategies investigated that are focused on improving gut health in pigs, prebiotics and probiotics are clear nutritional options. This review aims to summarize the current knowledge of the beneficial effects of probiotic microorganisms with the capacity to modulate the immune system (immunobiotics) in the regulation of intestinal inflammation in pigs. We discuss the role of toll-like receptors (TLRs), their signaling pathways, and their negative regulators in both the inflammatory intestinal injury and the beneficial effects of immunobiotics in general, and *Lactobacillus jensenii* TL2937 in particular. This review article emphasizes the cellular and molecular interactions of immunobiotics with intestinal epithelial cells (IECs) and immune cells through TLRs and their application for improving animal health and also human’s because the pigs are expected to be a better human model than rodents.

## TLR4 Signaling Pathway and Inflammation in the Gut

Toll-like receptor-4 is expressed by epithelial and immune cells and might play a role in the intestinal mucosal host defense against Gram-negative bacteria. However, since many body surfaces are colonized by the physiological microflora, activation of epithelial TLR4 must be tightly controlled to avoid unintended stimulation and mucosal inflammation.

**Figure 1:**
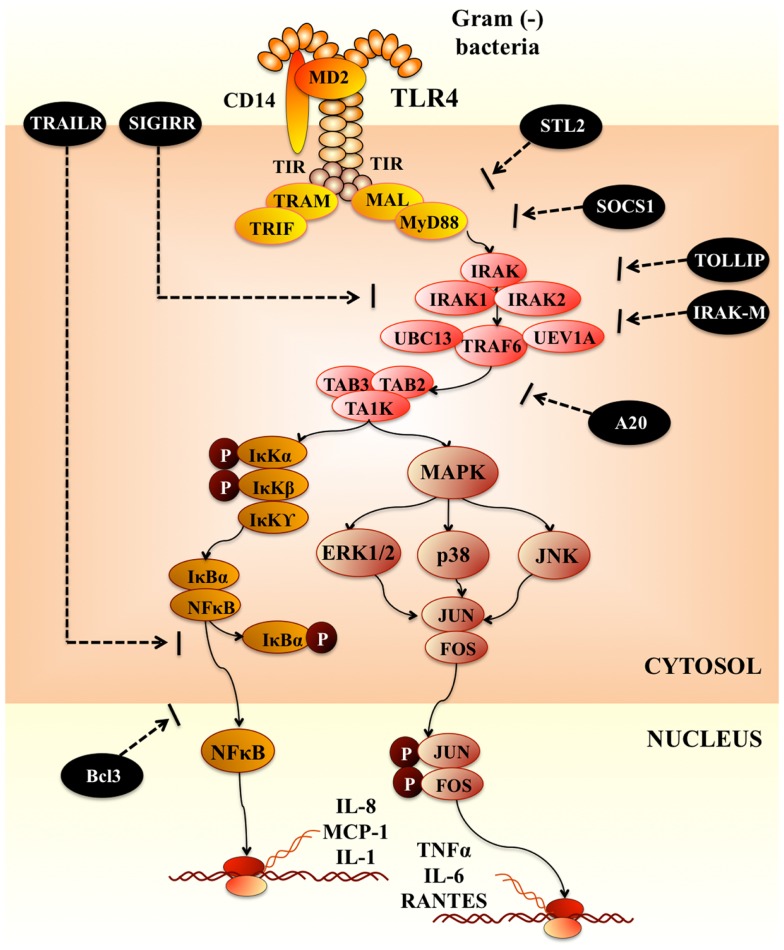
**Toll-like receptor-4 signaling pathway**.

Upon recognition of its cognate ligand, TLR4 dimerizes and initiates a signaling cascade that leads to the activation of a pro-inflammatory response (Figure 1). Ligand binding can induce two signaling pathways, the myeloid differentiation primary response gene 88 (MyD88)-dependent and MyD88-independent pathways, which induce the production of pro-inflammatory cytokines and type I IFNs (12). These two distinct responses are mediated via the selective use of adaptor molecules recruited to the TIR domains of the TLRs after ligand recognition and binding. Four adaptor molecules have been identified so far: MyD88, TIR-associated protein (TIRAP), TIR domain-containing adaptor protein-inducing IFN-β (TRIF), and TRIF-related adaptor molecules (TRAM) (13). MyD88 and TIRAP are responsible for the induction of pro-inflammatory genes, and TRIF and TRAM induce IFNs. In MyD88-dependent signaling, upon ligand recognition, MyD88 is recruited to and associates with the cytoplasmic domain of the TLRs. Then IL-1R-associated kinase 4 (IRAK-4) and IRAK-1 are recruited and activated by phosphorylation. Activated IRAK-4 phosphorylates IRAK-1, which subsequently associates with tumor necrosis factor receptor (TNFR)-associated factor 6 (TRAF6). TRAF6 activates transforming growth factor (TGF)-activating kinase 1 (TAK1) (Figure 1). TAK1 phosphorylates IKK-b and mitogen-activated protein kinase (MAPK) kinase 6 (MKK6), leading to degradation of I-κB and thereby leading to the nuclear translocation of NF-κB, which results in the induction of genes involved in inflammatory responses (Figure 1). Activation of the MyD88-dependent pathway also results in the activation of MAPKs such as p38 and JNK, which leads to the activation of AP-1 (13). For the MyD88-independent signaling TLR4 activation triggers the induction of a type I IFN response, leading to the induction of IFN-α and IFN-inducible genes.

Various negative regulatory mechanisms have evolved to attenuate TLR signaling and maintain the immune balance. At least six levels of negative regulation have been discovered so far (14, 15): (i) degradation of TLRs; (ii) down-regulation of transcription of TLRs and related genes; (iii) post-transcriptional repression by microRNAs (miRNAs); (iv) production of soluble TLRs functioning as decoy receptors; (v) intracellular inhibitors and; (vi) membrane-bound suppressors that inhibit TLR signaling pathways after TLR and ligand interactions have occurred. TLR signaling pathways can be tightly regulated by transmembrane proteins ST2, single immunoglobulin interleukin-1-related receptor (SIGIRR), and TNF-related apoptosis-inducing ligand receptor (TRAILR). SIGIRR is an orphan receptor that does not induce NF-κB activation. It interacts with IRAK and TRAF6 and inhibits TLR signaling. In contrast, TRAILR suppresses NF-κB activation at downstream TLR signaling events, perhaps by stabilizing IκB and preventing its degradation (14) (Figure 1). Another important negative regulatory mechanism for TLR signaling involves the endogenous intracellular negative regulators such as sMyD88 (the short form of MyD88), interleukin-1 receptor-associated kinase M (IRAK-M), suppressor of cytokine signaling 1, NOD2, phosphatidylinositol 3-kinase, Toll interacting protein (TOLLIP), and A20. The intracellular negative TLR regulators can act at multiple levels. For example, IRAK-M can heterodimerize with IRAK-1 or -2 and bind both MyD88 and TRAF6. Upon TLR-TIR-ligand engagement and formation of the MyD88 adaptor complex, IRAK-M is thought to bind MyD88/IRAK-4 and inhibit IRAK-4 phosphorylation of IRAK-1. This prevents formation of TRAF6/IRAK-1 complexes, which initiate IκB kinase and MAPK signaling pathways (16) (Figure 1). Some intracellular regulators are constitutively expressed to control TLR activation at a physiological level, whereas others are up-regulated by TLR signaling during infection to attenuate the TLR response in a negative feedback loop. Therefore, regulation of TLR signaling pathways constitutes a complex network.

Toll-like receptor signaling in IECs and immune cells has been shown to be involved in three important mechanisms that are crucial for maintaining a healthy epithelial barrier: (i) epithelial cell proliferation and maintenance of tight junctions; (ii) expression of antimicrobial factors; and (iii) modulation of immune responses [reviewed in Ref. (15)]. In a healthy individual, intestinal colonization stimulates these mechanisms that in turn contain the microbiota within the intestinal lumen and neutralize MAMPs. Moreover, these mechanisms protect the host from the systemic translocation of bacteria or bacterial products and from the outburst of pro-inflammatory cascades in intestinal epithelial and innate cells (17).

## Modulation of Intestinal Inflammation by Commensal Bacteria

Several studies have identified a role for pattern-recognition receptors (PRRs) in mediating non-inflammatory immune responses to the microbiota, challenging the paradigm that PRRs have evolved solely to recognize and respond to pathogens. MyD88-deficient mice are more susceptible to DSS-induced colitis, suggesting that commensal bacteria may be directly recognized by TLRs under steady-state conditions to mediate host-protective responses (18). To corroborate this notion, depletion of gut bacteria with antibiotics results in increased susceptibility to DSS; remarkably, oral feeding of lipopolysaccharide and lipoteichoic acid corrects this predisposition to colitis, revealing that TLR ligands have beneficial effects on the host (18). As DSS induces intestinal injury, these findings suggest that TLR signaling by the microbiota leads to maintenance of intestinal epithelial homeostasis in the absence of enteric pathogens. The polysaccharide from *Bacteroides fragilis* is a unique TLR2 ligand found in the human microbiome, which orchestrates anti-inflammatory immune responses that ameliorate diseases mediated by the immune system. This polysaccharide is ingested by intestinal DCs, which then stimulate responses of Foxp3^+^ Treg cells (19). Interestingly, TLR2-deficient mice are not protected by the polysaccharide against colitis (20). TLR2-deficient DCs do not promote responses of Foxp3^+^ Treg cells and production of IL-10, demonstrating that specific gut bacterial molecules have evolved to promote benefits to the host via PRR signaling in antigen presenting cells. Studies have also demonstrated that commensal organisms may target and inhibit NF-κB activation to suppress inflammation. By analyzing the composition of the intestinal microbiota of Crohn’s disease patients, Sokol et al. (21) identified *Faecalibacterium prausnitzii*, which is greatly reduced in Crohn’s disease patients, as an anti-inflammatory commensal bacterium in the gut by showing that the supernatant of *F. prausnitzii* inhibits NF-κB activation in a human IEC line and suppresses the production of pro-inflammatory cytokines both *in vitro* and in a mouse colitis model. However, the molecular mechanism by which this process occurs was not elucidated. Several studies have also highlighted the importance of TLR-MyD88 signaling among lymphocytes. In B cell–specific MyD88-deficient mice, bacteria disseminate to systemic sites, such as liver or lung, after DSS-induced damage of the colon, but not in epithelial cell–specific or dendritic cell–specific MyD88-deficient mice (22). Further, it has recently been appreciated that T cell subsets express functional TLRs (23). Transfer of MyD88-deficient T cells into RAG-deficient mice results in less intestinal inflammation (24). Conversely, whereas TLR signaling by T cells was classically thought to promote immunity, it now appears that this process can restrain inflammatory responses. For example, treatment of CD4^+^ T cell subsets with a TLR4 agonist increases suppressive activity and enhances protection from colitis (25). Therefore, TLRs represent a dynamic signaling system that triggers various immune outcomes, and TLR signaling directly by adaptive immune cells mediates reactions in the absence of innate immune cells.

A growing list of inhibitors for TLR signaling in the intestinal mucosa, including IRAK-M, TOLLIP, SIGIRR, A20, and peroxisome proliferator-activated receptor-γ (PPARγ), ensure that chronic inflammatory and potentially destructive TLR responses to MAMPs do not occur (26). In this regard, IECs deficient in SIGIRR are more susceptible to commensal-dependent intestinal inflammation, indicating that the intrinsic expression of SIGIRR by IECs regulates the communication between commensal bacteria and the host immune system (27). Additionally, an anti-inflammatory mechanism activated by commensal *B. thetaiotaomicron* that attenuates pro-inflammatory cytokine expression in IECs by promoting nuclear export of the NF-κB subunit RelA through a PPARγ-dependent pathway has been reported (28). Furthermore, the contact time between IECs and commensal bacteria seems to be critical, as short-term stimulation with LPS leads to activation of pro-inflammatory signaling cascades in IECs, including phosphorylation of IRAK and MAPK and increased IL-8 secretion, whereas prolonged incubation results in a state of hyporesponsiveness with minimal reaction by the IECs. Up-regulation of inhibitory TOLLIP contribute to this hyporesponsiveness (29).

In addition to TLRs, other PRRs have been involved in the anti-inflammatory effects of gut microbiota. The peptidoglycan recognition protein (PGRP) family is involved in the regulation of commensal microbiota in mice. Mice deficient in any one of the four PGRPs harbor a microbiota that promote increased sensitivity to DSS-induced colitis (29). Indeed, germ-free mice inoculated with stool from PGRP-deficient donor mice are more sensitive to DSS-induced colitis compared to mice that received stool from wild-type mice and exhibit greater mortality, weight loss, and colitis scores. Thus, mammalian PGRPs are important in shaping a homeostatic commensal microbiota and preventing intestinal inflammation (29). It is probable that in the near future studies will demonstrate that other PRRs are involved in the complex bidirectional cross-talk between commensal gut bacteria and the host.

## Modulation of Intestinal Inflammation by Probiotic Bacteria

Several studies have shown that immunobiotics can beneficially modulate the PRRs-mediated inflammatory response in the gut by modulating the functions of IECs and APCs (30, 31).

Probiotics inhibit excessive NF-κB-induced pro-inflammatory cytokine production by IECs. Immunobiotics suppress TNF- or *S. typhimurium*-induced IL-8 gene expression and secretion by IECs in an NF-κB-dependent manner (32, 33). A study in Caco-2 cells demonstrated that *Lactobacillus rhamnosus* GG counteracts the enterotoxigenic *Escherichia coli* (ETEC)-induced up-regulation of IL-1β and TNF-α and the down-regulation of TGF-β1 expression, consequently blocking cytokine deregulation (30). In addition, comparative studies between *L. rhamnosus* GG and *Bifidobacterium animalis* MB5 demonstrated that individual strains of probiotics have a different impact on the inflammatory response triggered in IECs (30). Others studies evaluating the effect of immunobiotic yeasts have shown that *Saccharomyces cerevisiae* CNCM I-3856 decreases the expression of the pro-inflammatory mediators IL-6, IL-8, CCL20, CXCL2, and CXCL10 in porcine intestinal epithelial IPI-2I cells cultured with F4^+^ ETEC (34). Moreover, the CNCM I-3856 strain inhibits ETEC-induced expression of pro-inflammatory cytokines and chemokine transcripts and proteins, and this inhibition is associated with a decrease in ERK1/2 and p38 MAPK phosphorylation and an increase in the mRNA level of anti-inflammatory PPARγ (35).

Additionally, the importance of direct stimulation of DCs by immunobiotics to promote tolerance was illustrated by some studies. Comparative studies using *Lactobacillus plantarum* NIZO B253, *Lactobacillus casei* NIZO B255, and *Lactobacillus reuteri* ASM20016 showed that *L. reuteri* and *L. casei*, in contrast to *L. plantarum*, prime DCs to promote the development of Treg cells. Experiments with TLR transfectants showed that none of the three lactobacilli tested substantially activated TLRs. However, *L. reuteri* and *L. casei* both potently induce the development of Treg cells and are recognized by DC-SIGN on DCs, an interaction that appears to be crucial for the priming of regulatory DCs (36). Another study showed that the direct interaction between DCs and *Lactobacillus acidophilus* NCFM is sufficient to induce IL-10 production and low IL-12p70 production by these cells. This acquisition of a non-inflammatory phenotype by the DCs was dependent on the activation of DC-SIGN that recognizes surface layer protein A (SlpA) of the bacterium. *L. acidophilus* with mutated SlpA fails to induce Th2 polarization of the DCs, and instead, promotes IL-12p70, TNF-α, and IL-1 production (37). Additionally, it was reported that transfer of LAB-treated bone-marrow-derived DCs protects mice from 2-4-6-trinitrobenzenesulfonic acid-induced colitis. This effect is mediated by TLR2 and NOD2 activation of the DCs and depends on the activation of Treg cells (38). Teichoic acid, a cell wall component of the Gram-positive bacteria *L. plantarum* NCIMB8826, is involved in the anti-inflammatory activity of this strain. A mutant with enhanced anti-inflammatory capacity incorporates much lower levels of d-Ala in its teichoic acids than the wild-type strain and induces dramatically reduced secretion of pro-inflammatory cytokines by blood monocytes, resulting in a significant increase in IL-10 production. The effects observed were clearly TLR2 dependent. This mutant was also more protective in a murine colitis model than its wild-type counterpart (39). Some probiotics activate anti-inflammatory and regulatory immune effects in the settings of enteric infections and mucosal inflammation. *Lactobacillus paracasei* CNCM I-4034 and its supernatant dramatically reduce the production of IL-6, IL-8, IL-12p70, and TNF-α in human intestinal DCs challenged with *Salmonella typhi* (40). These authors demonstrated that *L. paracasei* CNCM I-4034 activates the expression of TLR2 in DCs, up-regulates the expression of TOLLIP, and promotes the stimulation of TGF-β2, whereas the supernatant of the probiotic increases the secretion of TGF-β1.

Lebeer et al. (41) suggested that the final outcome of a host cell response against probiotic bacteria depends on the combination of the distinct MAMPs that can interact with the various PRRs and associated co-receptors that fine-tune signaling; as well as on the concentration of these MAMPs. To date, several MAMPs of immunobiotics have been indentified, that can be connected to specific host responses (41) and these effector molecules are in many cases associated with the bacterial cell surface (42). Although most beneficial effects of probiotics require direct bacterium-cell contact with live bacteria, some reports demonstrated that soluble factors secreted by probiotics are able to modulate the production of cytokines and therefore, to modulate the immune system. In fact, recent investigations have exposed some of the underlying mechanisms in the modulation of gut immune system by probiotic soluble factors. Peña and Versalovic (43) reported that *L. rhamnosus* GG specifically inhibits TNF-α production and reduces TNF-α/IL-10 ratios in a murine macrophage model with an anti-inflammatory net effect. This effect is contact-independent, requiring the presence of a soluble *L. rhamnosus* GG immunomodulin for complete modulatory activity. The putative immunomodulin has a protein or peptide component that inhibits TNF-α production in murine macrophages. Further research work using *L. rhamnosus* GG strain, to investigate molecular mechanisms by which probiotics regulate IECs, reported the purification of two novel *L. rhamnosus* GG-derived soluble proteins, p75 and p40. Each of these purified protein preparations activated Akt, inhibited cytokine-induced epithelial cell apoptosis, and promoted cell growth in human and mouse colon epithelial cells and cultured mouse colon explants. TNF-induced colon epithelial damage was significantly reduced by p75 and p40. Immunodepletion of p75 and p40 reversed the *L. rhamnosus* GG conditioned media activation of Akt and its inhibitory effects on cytokine-induced apoptosis and loss of IECs (44). These findings suggest that probiotic bacterial components may be useful for preventing cytokine-mediated gastrointestinal diseases. Another example of a secreted protein associated with probiotic activity is the *prt-P*-encoded protease of *L. paracasei* that degrades secreted CXCL10 (also called IP-10), resulting in reduced lymphocyte recruitment in an ileitis model (45). Secreted factors produced by *Lactobacillus casei-rhamnosus* were tested on human lymphocytes, monocytes, and a human monocytic leukemia-cell line (THP-1). The soluble factor(s) present in supernatants effectively induced apoptosis of immune cells. These were mainly soluble heat-stable proteins. For immune cells, pre-treatment with the supernatant significantly promoted apoptosis via a mitochondrial pathway. The supernatant also inhibited the release of LPS-induced pro-inflammatory cytokines TNF-α, IL-1β, IL-6, and IL-8 by immune cells (46). It was also described that in the human gut, *L. plantarum* secretes an extracellular protein that releases an internal fragment (STp) when is cleaved by intestinal proteases. It is characterized by the abundance of serine and threonine residues within its sequence. STp is encoded in one of the main extracellular proteins produced by such species, which includes some probiotic strains. *In vitro* studies using DCs from human peripheral blood showed that STp increased the production of regulatory IL-10 in healthy controls. In addition, T cells stimulated with STp-pulsed DCs decreased the production of pro-inflammatory IFNs and increased anti-inflammatory IL-10 production, suggesting that these T cells acquired an immunoregulatory phenotype (47).

## Intestinal Inflammation in Piglets after Weaning: Impact of Probiotics in Immune Health and Productivity

The weaning transition is a complex period during which the piglets have to face an abrupt separation from their mother, mixing with other litters in a usually new environment, and switch from milk to a solid feed which involves a change from a highly digestible to a less-digestible and more-complex feed. In consequence, several physiological changes occur in the intestine of pigs during the process of weaning [reviewed in Ref. (11)]. Early studies of Pluske et al. (48) showed that weaning induces several modifications in the intestinal tissue including changes in villus and crypt architecture and reduced activities of brush-border digestive enzymes. Moreover, these histological and physiological modifications have been implicated in a higher susceptibility to intestinal pathogens such as *E. coli* and rotaviruses (48). Changes in the gut microbiota have been also described. The gut of piglets is sterile at birth and is then colonized by microbes from the mother and the environment, starting with lactic acid bacteria, enterobacteria, and streptococci. After weaning and the introduction of solid feed obligate anaerobes increase in number and diversity until an adult-type pattern is achieved (11, 49). These modifications in microbial communities has a great impact in the gastrointestinal health of piglets, considering that microbial activity is important for improvement of energy yield, vitamin production, fermentation of carbohydrates, gut motility, production of volatile fatty acids, and water and Na^+^ absorption [reviewed in Ref. (11)].

In addition, it has to be considered that the piglet is not immunocompetent at birth. Piglet is dependent on a supply of several specific and non-specific immune factors present in maternal colostrum and milk for immune protection, resistance against pathogens, development, and survival. Clearly, development of immunocompetence is an absolute requirement for optimum growth and performance. Early weaning at 3 weeks of age is associated with a transient reduction in the ability of intraepithelial lymphocytes to respond to mitogens and splenic T cells to secrete IL-2. Furthermore, tolerance to fed proteins introduced at weaning is not fully achieved until 8 weeks of age (11).

A growing body of experimental and clinical evidence supports the therapeutic and preventive application of probiotics for several gastrointestinal inflammatory disorders in pigs. In this regard, Qiao et al. (50) conducted experiments to evaluate the effects of a complex Lactobacilli preparation on performance, resistance to *E. coli* infection and gut microbial flora of weaning pigs. The mix of four lactobacilli (*Lactobacillus gasseri, L. reuteri, L. acidophilus*, and *Lactobacillus fermentum*) isolated from weaning pigs was able to reduce *E. coli* and anaerobe counts in the gut, and decrease diarrhea. Additionally, lactobacilli treatment significantly improved average daily feed intake of pigs compared to controls during the first 2 weeks after weaning and the average daily gain (50). It is known that the ratio of Bacteroidetes and Firmicutes bacterial groups in the gut can affect the ability to absorb nutrients. Therefore, Cui et al. (51) investigated the effect of probiotic *Bacillus subtilis* on Bacteroidetes and Firmicutes in cecal contents and growth performance and fat deposition in weaning piglets. The study found that the addition of *B. subtilis* improves growth performance and affects lipid metabolism through regulation of the proportion of Bacteroidetes and Firmicutes in the gut. Herfel et al. (52) examined the impact of a novel probiotic strain of *Bifidobacterium longum* AH1206 on the health, growth, and development of neonatal pigs. Authors found that ileal IL-10 expression increased progressively with AH1206 supplementation, which indicated the potential for modulation of the inflammatory tone of the intestinal mucosa of suckling piglets. However, no differences were found between AH1206-treated and control piglets when comparing body weight gain, feed efficiency (gain:intake), and histological and physiological modifications in intestines. Another recent study evaluated the effect of the co-administration of *B. subtilis* RJGP16 and *Lactobacillus salivarius* B1 on intestinal immunity in piglets (53). Authors demonstrated that probiotic administration increased the expression of IL-6, porcine beta-defensins, and IgA producing cells in the intestine, clearly showing that co-administration of RJGP16 and B1 strains strongly enhances the intestinal mucosal immunity of piglets.

Some recent studies have specifically evaluated the capacity of probiotics to improve the resistance of piglets against ETEC. It was shown that the probiotic strain *L. plantarum* CJLP243 may serve as a potential alternative to antibiotic supplementation to improve the growth and health performance of weaning pigs because of its capacity to reduce the severity of ETEC-induced diarrhea (54). Li et al. (55) showed that pre-treatment of piglets with *L. rhamnosus* ATCC7469 ameliorates F4^+^ETEC-induced diarrhea. In piglets exposed to F4^+^ETEC, jejunal TLR4 and IL-8 expression were increased; however, these increases were attenuated by administration of *L. rhamnosus*. Notably, expression of jejunal TLR2, ileal TLR9, NOD1, and TNF-α was up-regulated in the ATCC7469-treated piglets after F4^+^ETEC challenge (55). These results indicate that probiotic treatments would be able to beneficially modulate the overwhelming inflammatory response in infected piglets.

Although these studies demonstrated that is possible to modulate piglets’ gut microbiota and immunity and improve growth performance by using appropriate probiotics strains, the true efficacy of probiotics in agricultural animals remains unclear because of inconsistent experimental results. Explanations for the disparities between studies include differences in experimental conditions, animal age, genetics, and health status. Additionally, the inconsistent results could be attributed to a lack of understanding of detailed cellular and molecular mechanism of action, as well as unknown interactions among these bacteria, the host, and the intestinal microbiota (56).

## Modulation of TLR4-Mediated Inflammation in Intestinal Epithelial Cells by *Lactobacillus jensenii* TL2937

**Figure 2:**
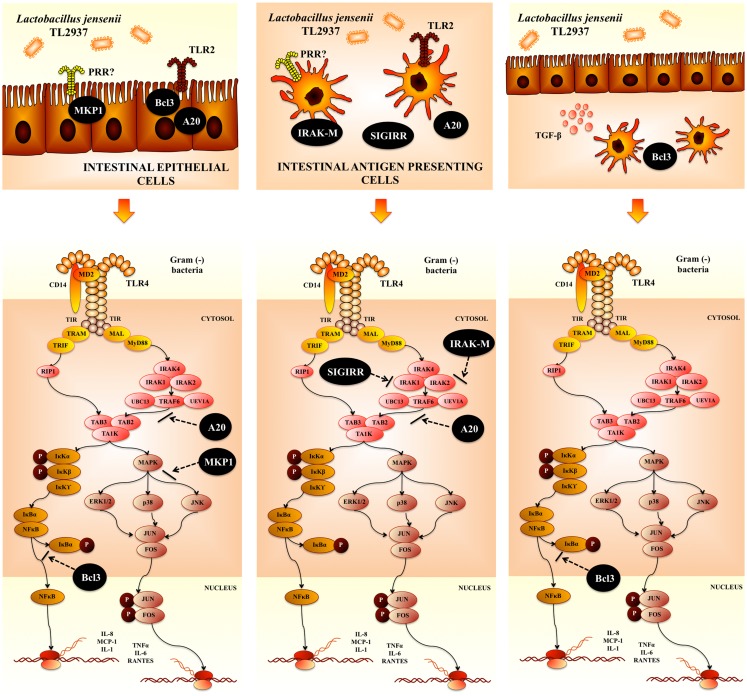
**Modulation of negative regulators of toll-like receptor-4 signaling pathway by *Lactobacillus jensenii* TL2937 in porcine intestinal epithelial cells and antigen presenting cells**.

Intestinal epithelial cells are a central component of the immune system of the gut. Several works have demonstrated that microbial recognition by IECs is an integral aspect of first-line host responses. Then, current observations point to the idea that more than simply a physical barrier separating luminal contents from mucosal APCs, the intestinal epithelium is increasingly recognized as playing an essential role in immune homeostasis, through the promotion of tolerogenic and regulatory responses. These findings have important implications for the regulation of mucosal homeostasis by probiotic bacteria. To study the mechanisms by which IECs induce an immune response to pathogens and the potential immunoregulatory effect of immunobiotics in pigs, we established a clonal porcine intestinal epitheliocyte cell line (PIE cells) (58). Studies of TLRs expression in PIE cells demonstrated that TLR4 is expressed most strongly. It was confirmed that PIE cells, which preferentially express TLR4/MD-2, undergo inflammatory responses regarding cytokine expression in response to LPS stimulation (58). Moreover, stimulation of PIE cells with porcine-specific ETEC significantly increases the levels of IL-6, IL-8, and monocyte chemotactic protein (MCP)-1 (57). It was also found that damage to PIE cells correlates with the levels of pro-inflammatory cytokines produced after stimulation with ETEC and LPS (57), which is consistent with reports demonstrating that challenging human intestinal Caco-2 cells with ETEC causes strong up-regulation of pro-inflammatory mediators that lead to membrane damage (59, 60). We selected lactobacilli strains that regulate the inflammatory response induced by ETEC and LPS in PIE cells by evaluating the levels of IL-1α, IL-6, IL-8, and MCP-1. The challenge of PIE cells with the intestinal pathogen significantly increased levels of pro-inflammatory cytokines in lactobacilli-untreated control cells (61). However, IL-6 and IL-8 levels in PIE cells stimulated with some lactobacilli strains, especially *L. jensenii* TL2937, were significantly lower than those in the control (61). Interestingly, *L. jensenii* TL2937, a strain with a high capacity to activate TLR2, was also the strain with the highest capacity to down-regulate IL-6 and IL-8 production by PIE cells in response to ETEC and LPS. For this reason, we became interested in *L. jensenii* TL2937 and examined the mechanisms behind the anti-inflammatory effect mediated by this strain, and demonstrated that *L. jensenii* TL2937 inhibits NF-κB and MAPK signaling pathways in ETEC- and LPS-challenged PIE cells (Figure 2).

Proteins that regulate the intensity and duration of TLR activation are able to modulate the cellular outcome, thereby controlling whether TLR activation leads to homeostatic or inflammatory responses (14). To dissect the mechanism(s) involved in the anti-inflammatory effect of *L. jensenii* TL2937, the effect of this strain on the expression of the negative TLR regulators in PIE cells was evaluated. The expression of SIGIRR, Tollip, A20, Bcl-3, MKP-1, and IRAK-M was studied, and it was found that MKP-1, A20, and Bcl-3 mRNA expression was up-regulated in PIE cells stimulated with *L. jensenii* TL2937 (61) (Figure 2). MKP-1 plays a role in the inhibition of pro-inflammatory mRNA expression by inactivating MAPK. MKP-1 desensitizes cells to TLR ligands by inactivating the p38 signaling pathway in enterocytes (62). Moreover, MKP-1 is not induced by TLR2 stimulation, although ligands for TLR3, TLR4, TLR5, and TLR9 induce MKP-1. This is in agreement with our finding that the TLR2 ligand Pam3CSK4 itself does not induce the expression of MKP-1 (61). Bcl-3 functions as an inhibitor of NF-κB activity by stabilizing repressive NF-κB homodimers in a DNA-bound state and preventing the binding of transcriptionally active dimers. In fact, stabilization of repressive complexes through the induction of Bcl-3 expression has been proposed to function during the processes of LPS tolerance (63). Moreover, treatment of macrophages with IL-10 induces the expression of Bcl-3, leading to inhibition of LPS-induced TNF-α production (64).

*Lactobacillus jensenii* TL2937 also upregulate the expression of A20 in PIE cells. A20 is a zinc finger protein that inhibits activation of NF-kB via inflammatory cytokine receptors (65, 66), TLR (67, 68), and the nucleotide-binding oligomerization domain-containing receptor NOD2 (69). A20 functions via its two ubiquitin-editing activities, an N-terminal deubiquitinase that removes K63-linked polyubiquitin chains and a C-terminal ubiquitin ligase that facilitates target protein degradation via attachment of K48-linked poly-ubiquitin chains (66, 70). These two activities cooperatively down-regulate TRAF6 (71). Therefore, A20 plays an essential role in the termination of NF-kB signaling in response to TNF-α and microbial products such as LPS (72). A20 deficiency in enterocytes renders mice sensitive to TNF-a-induced lethal inflammation, leading to disruption of the epithelial barrier and infiltration of commensal bacteria that initiate a systemic inflammatory response (73). These data suggest that A20 is important for the inhibition of innate immune responses in the gut (26). In addition, gut decontamination with a mixture of antibiotics with limited oral bioavailability in drinking water markedly reduces A20 protein and mRNA levels in the ileal epithelium of mice (74). Moreover, partial rather than complete abrogation of A20 expression is likely due to incomplete elimination of intestinal bacteria by the antibiotic treatment (74). These results show that A20 expression in the epithelium positively correlates with the bacterial load in the lumen. The observations that A20-deficient mice develop severe gut inflammation early in life (75) and that this inflammatory state can be alleviated by antibiotics or knockout of the TLR signaling mediator myeloid differentiation factor MyD88 (76) further support a key role for A20 in intestinal tolerance to the intestinal microbiota.

Recently, it was demonstrated that *B. longum* BB536 and *Bifidobacterium breve* M-16V significantly down-regulated levels of IL-8, MCP-1, and IL-6 in PIE cells challenged with ETEC by modulating the NF-kB and MAPK pathways (77). Moreover, both bifidobacteria up-regulated A20 in PIE cells. Then, the most effective anti-inflammatory strains evaluated in our laboratory, *L. jensenii* TL2937 and bifidobacteria strains BB536 and M-16V, strongly up-regulated the ubiquitin-editing enzyme A20. This finding is of interest because it not only shows a common mechanism for the anti-inflammatory activity of immunobiotics but also provides a potential biomarker for the screening and selection of new immunoregulatory strains.

## Modulation of TLR4-Mediated Inflammation in Intestinal Antigen Presenting Cells by *Lactobacillus jensenii* TL2937

Considering the anti-inflammatory effects of the TL2937 strain in IECs and the critical importance of APC polarization in immunoregulation, it was also examined the effect of *L. jensenii* TL2937 on activation patterns of APCs from porcine Peyer’s patches (PPs). In swine, the most frequent marker expressed on DCs and macrophages is CD172a. Additionally, CD11R1 is considered to be a marker that is specifically and differentially expressed on porcine DCs, but not on macrophages (78). Then, in our studies we used CD172a and CD11R1, together with MHC-II, to define three different populations of APCs in porcine PPs: CD172a^+^CD11R1^high^, CD172a^−^CD11R1^low^, and CD172a^+^CD11R1^−^ cells (79). According to our studies and previously published works (72, 80, 81), CD172a^+^CD11R1^high^ and CD172a^−^CD11R1^low^ cells could be DCs, and CD172a^+^CD11R1^−^ cells could be macrophages; however, functional studies are needed to accurately define each population. Therefore, in our studies, we refer to each of the three populations as APCs.

*Ex vivo* experiments using the adherent population of PPs APCs showed that the treatment with *L. jensenii* TL2937 increases the expression of IL-10 and TGF-β in CD172a^+^CD11R1^high^ and CD172a^+^CD11R1^−^ cells, whereas treatment with this bacterium is associated with increased levels of IFN-γ in CD172a^−^CD11R1^low^ cells (79). Then, the direct exposure of porcine APCs to *L. jensenii* TL2937 in the absence of inflammatory signals activates CD172a^+^ APCs and causes them to become phenotypically and functionally mature and to display tolerogenic properties (79). Our findings show similarities to previous studies with lactobacilli and APCs from different species. For example, human myeloid DCs exposed to lactobacilli show increased expression of MHC-II and co-stimulatory molecules (39, 82, 83). Moreover, similar to our work, previous studies by Drakes et al. (36) reveal that probiotic lactobacilli induce up-regulation of IL-10 production and cell surface markers of maturation and activation in DCs (36).

On the contrary, *L. jensenii* TL2937 increased the production of IFN-γ in CD172a^−^CD11R1^low^ cells (79). One possible explanation for the differential immunoregulatory effect of TL2937 may be the levels of expression of TLR2 in distinct APCs. CD172a^+^CD11R1^high^, CD172a^+^CD11R1^−^, and CD172a^−^CD11R1^low^ cells differ regarding TLR2 expression (79), and therefore, they are likely to differ in the degree to which they interact with *L. jensenii*. In support of this hypothesis, it was reported that teichoic acid, a cell wall component of the Gram-positive bacteria *L. plantarum* NCIMB8826, is involved in the anti-inflammatory activity of this strain. A mutant with enhanced anti-inflammatory capacity incorporates much lower levels of d-Ala in its teichoic acids than the wild-type strain and induces dramatically reduced secretion of pro-inflammatory cytokines by blood monocytes, resulting in a significant increase in IL-10 production. The effects observed were clearly TLR2 dependent. This mutant was also more protective in a murine colitis model than its wild-type counterpart (37). Other PRRs would be also involved in the immunoregulatory effect of immunobiotics on APCs. Comparative studies using *L. plantarum* NIZO B253, *L. casei* NIZO B255, and *L. reuteri* ASM20016 showed that *L. reuteri* and *L. casei*, in contrast to *L. plantarum*, prime DCs to promote the development of Treg cells. Experiments with TLR transfectants showed that none of the three lactobacilli tested substantially activated TLRs. However, *L. reuteri* and *L. casei* both potently induce the development of Treg cells and are recognized by DC-SIGN on DCs, an interaction that appears to be crucial for the priming of regulatory DCs (84). Another study showed that the direct interaction between DCs and *L. acidophilus* NCFM is sufficient to induce IL-10 production and low IL-12p70 production by these cells. This acquisition of a non-inflammatory phenotype by the DCs was dependent on the activation of DC-SIGN that recognizes SlpA of the bacterium (85).

Treatment of APCs with *L. jensenii* TL2937 also results in differential modulation of the production of pro- and anti-inflammatory cytokines in response to ETEC or LPS challenges. The differential effects of the TL2937 strain in each PPs APC population persist because increased production of IFN-γ is observed in CD172a^−^CD11R1^low^ cells and improved synthesis of IL-10 is detected in CD172a^+^CD11R1^high^ and CD172a^+^CD11R1^−^ cells (79).

In order to find the mechanism(s) involved in the immunoregulatory effects of the TL2937 strain, the expression of negative regulators of TLRs in porcine APCs was also evaluated. Of the six regulators tested, SIGIRR, A20, and IRAK-M mRNA expression was up-regulated in CD172a^+^ cells stimulated with *L. jensenii* TL2937 (Figure 2). It was shown *in vitro* that overexpression of SIGIRR inhibits TLR-induced NF-κB activation and attenuates the production of inflammatory cytokines (86). The LPS-induced inflammatory response is enhanced in SIGIRR-deficient mice (87). As described above, A20 also has an essential role in regulating inflammatory responses in the gut (68, 72). Notably, IRAK-M-deficient cells stimulated with TLR ligands or bacteria produce an increase in NF-κB and MAPK activation and elevated amounts of pro-inflammatory cytokines, such as IL-12, IL-6, and TNF-α (88). IRAK-M expression is induced upon LPS stimulation, and endotoxin tolerance is diminished in IRAK-M-deficient cells; these observations indicate that IRAK-M plays a critical role in regulating innate immunity through a negative feedback loop (89). Therefore, induction of these three negative regulators by *L. jensenii* TL2937 in CD172a^+^ APCs cells from swine PPs may be important for establishing tolerance to LPS and ETEC (Figure 2).

Although our studies in PIE cells and APCs demonstrated the ability of immunobiotics to modulate the inflammatory response, these *in vitro* models may be overly simplified and may not account for the effect of cell-cell interactions in a complex organic microenvironment, completely changing the resulting response. As mentioned before, IECs express a broad range of factors that may influence intestinal APCs and lymphocytes (90, 92). Therefore, to further assess the immunoregulatory effect of *L. jensenii* TL2937, in a recent study a co-culture system with a PIE cell monolayer and immunocompetent cells from swine PPs was used to model an *in vitro* PP culture system (91).

A significant up-regulation of pro-inflammatory cytokines was observed in PIE cells co-cultured with PPs APCs and challenged with ETEC or LPS. These results were consistent with findings described for PIE cells monocultures described above. Therefore, PIE cells did not responded differently to TLR4 activation when co-cultured with APCs (91). Moreover, it was confirmed that the pre-treatment of PIE cells with *L. jensenii* TL2937 reduced pro-inflammatory cytokines in response to ETEC or LPS and that this effect was related to up-regulation of the three TLR negative regulators: A20, Bcl-3, and MKP-1 as in PIE cell monocultures (61, 91, 93). In addition, *L. jensenii* TL2937-treated PIE cells were able to significantly upregulate TGF-β expression (91). It is well known that IECs-derived factors are able to condition mucosal DCs to secrete cytokines such as IL-10 and TGF-β in response to commensal microbes, thereby initiating differentiation of Treg immune responses (94). Moreover, conditioning of monocyte-derived DCs with IECs supernatants confer on DCs the capacity to produce large amounts of IL-10, which is attributable, at least in part, to the release of the IECs-derived factors such as TGF-β and thymic stromal-derived lymphopoietin (TSLP) (95). Therefore, in addition to its direct tolerogenic effects on PIE cells, *L. jensenii* TL2937 could have an indirect anti-inflammatory effect on APCs under the influence of factors produced by PIE cells such as TGF-β (91).

The study of the indirect effect of *L. jensenii* TL2937 on APCs in co-cultures, demonstrated that the response of these cells was completely different to those observed in APCs monocultures. In PIE-APCs co-cultures, no modifications in the levels of TGF-β in CD172a^+^CD11R1^−^ and CD172a^+^CD11R1^high^ cells or levels of IFN-γ in CD172a^−^CD11R1^low^ cells were observed. However, increased levels of IL-10 were found in CD172a^+^ cells co-cultured with PIE cells (91). In addition, no modification in SIGIRR, A20 or IRAK-M expression was observed in those cells. Notably, Bcl-3 expression was up-regulated in APCs cells co-cultured with PIE cells (91) (Figure 2). The Bcl-3 protein functions as an inhibitor of NF-κB activity. It was reported that treatment of macrophages with IL-10 induces the expression of Bcl-3, and Bcl-3 expression leads to inhibition of LPS-induced TNF-α production (64). Then it is probable that immunoregulatory cytokines (IL-10) produced by APCs act in an autocrine way and upregulate the expression Bcl-3. Then, the response of PPs APCs to *L. jensenii* TL2937 is significantly modified when the stimulus is mediated indirectly through IECs (91).

## Impact of *Lactobacillus jensenii* TL2937 in Pigs’ Immune Health and Productivity

Recent *in vivo* data concerning the immunoregulatory effect of *L. jensenii* TL2937 demonstrated that the administration of this immunobiotic strain improved immune health and growing performance and productivity of piglets (91). Feeding the TL2937 strain to 3 week-old LWD piglets significantly increased carcass grading (according to the standards of the Japanese Meat Grading Association) and improved juicy, tenderness, and overall palatability.

As mentioned before, at weaning, piglets are stressed, the food intake is strongly depressed, the structure and function of the gastrointestinal tract are altered, and these conditions can favor bacterial translocation, inflammation, and infection with pathogenic bacteria. It was reported that the optimal gut microbiota significantly improves intestinal health and beneficially affects the efficiency of gastrointestinal and whole body growth throughout the productive life cycle of a pig (11). In this regard, studies on the expression profiles induced by gut microbiota in the ileal epithelium of neonatal piglets showed and enhanced expression of NF-κBIA, a protein associated with the inactivation of NF-κB by sequestration, and the negative regulator of TLRs TOLLIP together with the down-regulation of GATA1 in colonized versus germ-free animals; reflecting the activation of pathways that prevent excessive inflammation. In addition, it is extremely important to direct piglets intestinal immune system toward appropriate immune responses that strives to maintain intestinal homeostasis, not only in the induction of tolerance against harmful antigens, but in effective effectors responses against pathogens. Some studies have associated probiotic bacteria with the improvement of intestinal homeostasis in pigs, albeit with different levels of success as described previously (53, 55). Considering the capacity of *L. jensenii* TL2937 to functionally modulate the response of PIE cells and porcine APCs, it was hypothesized that this strain would significantly impact on piglets’ immune heath. The *in vivo* experiments in pigs indicate that *L. jensenii* TL2937 is able to improve immunity and regulate excessive inflammation (91). These effects seem to be related to the complex secretion of cytokines induced by the probiotic strain in the gut. *L. jensenii* TL2937 could strongly induced secretion of IL-10 and IFN-γ that would be related to the beneficial effects achieved by the immunobiotic strain (91). The capacity to modulate inflammation and improve defenses at the same time has been described for several probiotic strains (95, 96). *L. jensenii* TL2937 could be included in the list of probiotic strains with those capabilities.

## Conclusion

Post-weaning diarrhea mainly occurs within the first week after weaning and affects pigs across the globe, causing great economic loss to the swine industry due to reduced growth performance and considerable morbidity and mortality. Our studies demonstrated that the use of immunobiotics strains as supplemental additives for piglet feedings could be used as a strategy to maintain and improve intestinal homeostasis; that is important for the development of the pig and for health and performance throughout the productive life of the animal.

**Figure 3:**
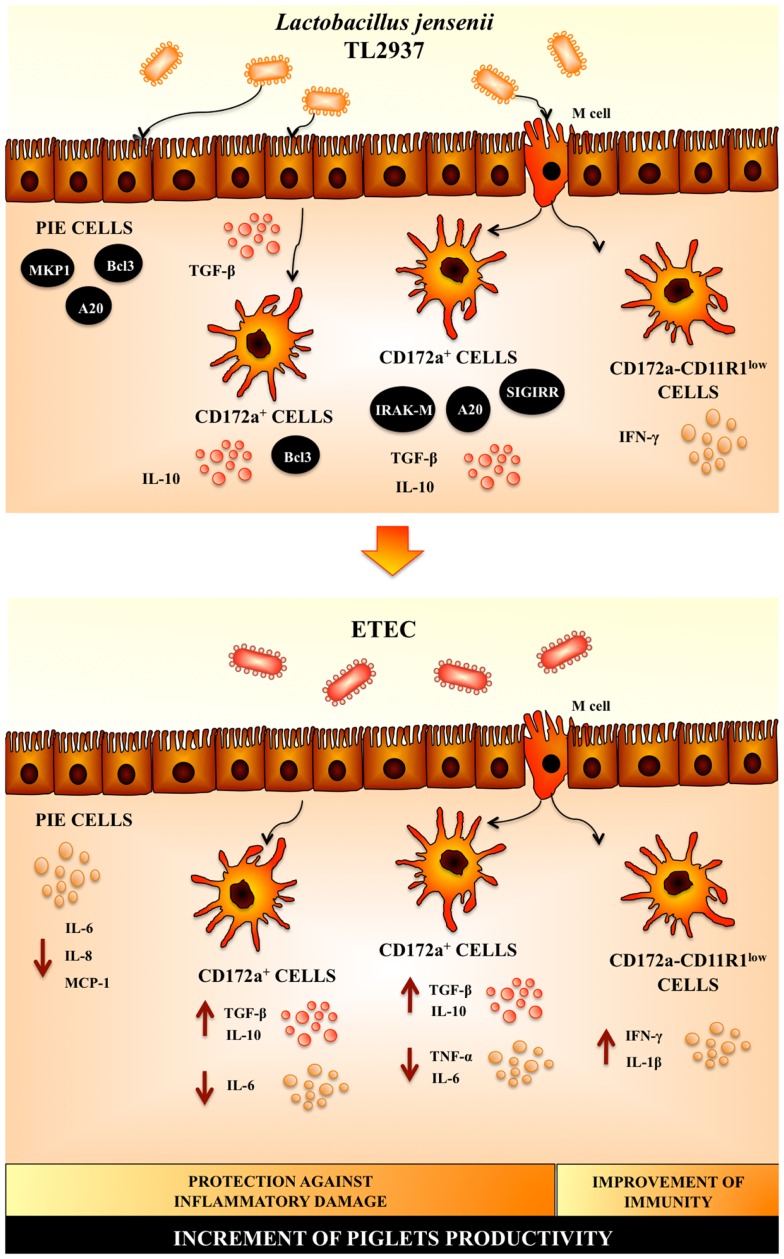
**Proposed mechanism for the immunoregulatory effect of *Lactobacillus jensenii* TL2937 in porcine intestinal mucosa**.

The scientific research into probiotic mode of actions has come to age and has shown how probiotics are able to induce beneficial changes in the host. Our research work allows us to propose a complete view of the cellular and molecular mechanisms involved in the immunoregulatory effects of *L. jensenii* TL2937 (Figure 3). When reaching the porcine intestinal mucosa, *L. jensenii* TL2937 would have the capacity to interact with local cells at three levels (Figure 3): (i) the interaction of the TL2937 strain with IECs would induce the up-regulation of MKP-1, Bcl-3, and A20 expression, which would be mostly dependent on TLR2 activation as we have demonstrated for several immunobiotic bacteria including the TL2937 strain; (ii) *L. jensenii* TL2937 could be taken by APCs indirectly through M cell transport or by direct sampling from the intestinal lumen, inducing an increase in the production of the immunoregulatory cytokines IL-10 and TGF-β by CD172a^+^ cells as well as the expression of SIGIRR, IRAK-M, and A20. In addition, through its direct interaction with CD172a^−^CD11R1^low^ cells, the TL2937 strain would have the capacity to improve Th1 responses by increasing the production of IFN-γ and; (iii) *L. jensenii* TL2937, through its capacity of stimulating the production of immunoregulatory factors such as TGF-β in EICs, would indirectly increase the expression of Bcl-3 and the production of IL-10 in CD172a^+^ APCs reinforcing its effects in these cells. Then, *L. jensenii* TL2937 would functionally modulated IECs and APCs to improve resistance against infections and avoid unproductive inflammation. In fact, experiments using ETEC challenge, clearly demonstrated that the TL2937 strain is able to induce protection against inflammatory damage and improve immunity at the same time (Figure 3). It was also demonstrated that the immunological networks induced by *L. jensenii* TL2937 help to maintain intestinal tolerance and improve the development of appropriate protective and controlled immune responses. Then, *L. jensenii* TL2937 has a great potential to be used as a pig probiotic feed. In addition, accumulation of empirical data in pigs may increase the probiotic use in human because the pigs are also expected for development as human model.

## Conflict of Interest Statement

The authors declare that the research was conducted in the absence of any commercial or financial relationships that could be construed as a potential conflict of interest.
